# The Course of Nasolabial Angle Loss After Rhinoplasty and Prediction of Late Outcome Using Machine Learning: A Proof-of-Concept Study

**DOI:** 10.3390/jcm15197469

**Published:** 2026-09-25

**Authors:** Sinan Seyhan, Levent Uğur, Mehmet Mustafa Erdoğan

**Affiliations:** 1 Department of Otorhinolaryngology and Head and Neck Surgery, Faculty of Medicine, Amasya University, Amasya 05100, Türkiye; snn.seyhan@hotmail.com; 2 Department of Mechanical Engineering, Faculty of Engineering, Amasya University, Amasya 05100, Türkiye; 3 Department of Otorhinolaryngology and Head and Neck Surgery, Kolmed Private Hospital, Amasya 05100, Türkiye

**Keywords:** rhinoplasty, nasolabial angle, nasal tip rotation, longitudinal tracking, machine learning, stabilization

## Abstract

**Background/Objectives**: The primary objective of this study was to predict the absolute nasolabial angle (NLA) at postoperative month 12 using early postoperative measurements. Secondary objectives were to characterize longitudinal NLA changes through month 12 and quantify residual angle loss. **Methods**: This single-center retrospective cohort study included 78 patients who underwent primary open rhinoplasty and had complete standardized lateral photographs from the preoperative assessment and postoperative months 1, 3, 6, and 12. The absolute NLA at month 12 was designated as the primary outcome and prediction target. Longitudinal NLA changes and residual loss from months 1, 3, and 6 to month 12 were secondary outcomes. NLA was measured using a Python (version 3.12.13)/OpenCV-based semi-automatic image-analysis system. Intraobserver and interobserver reliability were evaluated in a stratified random sample of 40 images measured twice by two physicians using intraclass correlation coefficients and Bland–Altman analysis. Linear and machine-learning models were evaluated using repeated nested cross-validation. Three simple benchmarks were also assessed using identical patient-level outer splits: the training-fold mean, persistence (NLA_12_ = NLA_6_), and linear regression using NLA_6_ alone. **Results**: NLA was highest at 1 month and decreased significantly thereafter, while remaining above the preoperative level at month 12. Measurement reliability was excellent, although measurement uncertainty was comparable to the mean late change. Elastic Net predicted absolute NLA_12_ with an MAE of 3.47°. It outperformed the training-mean and persistence benchmarks but did not outperform the one-predictor linear regression model (Mean Absolute Error (MAE) = 3.35°; paired MAE difference, 0.12°; 95% CI, −0.06 to 0.30). Patient-specific loss from months 6 to 12 could not be predicted reliably. **Conclusions**: The month-6 NLA provided moderate predictive information for the absolute 12-month angle. More complex multivariable algorithms did not provide additional predictive value over a simple regression based on NLA_6_ alone, and individualized late rotation loss remained unpredictable with the available variables.

## 1. Introduction

Rhinoplasty is one of the most challenging procedures in facial plastic surgery, reshaping the nose, the most prominent structure in the center of the face. The aim of rhinoplasty is to achieve a satisfactory result both aesthetically and functionally [1]. To this end, an attempt is made to create a nasal tip that is harmonious and balanced with the rest of the face and other parts of the nose. Even if the targeted nasal tip appearance is achieved in the early stages, changes can be observed over time due to processes such as edema resolution, soft tissue redraping, and scar maturation [2,3]. In particular, the long-term stabilization of nasal tip rotation can be a significant problem, causing dissatisfaction for both the patient and the surgeon, and necessitating revision surgery [2,4]. While regression in nasal tip rotation is expected in the long term, it may not always be possible to predict the extent and timing of this regression. The temporal pattern of nasal tip rotation and projection changes after rhinoplasty has not yet been clearly characterized.

Photogrammetric parameters such as the nasolabial angle (NLA) are commonly used in the objective assessment of nasal tip rotation [1,2,5]. The temporal effects of various surgical techniques on NLA have been reported. Studies with tip suturing techniques and various grafts have shown that tip rotation increases in the early postoperative period and then regresses somewhat; some techniques have less long-term rotation loss than others [6,7,8,9]. Although the structural support capacities of these techniques are proven and frequently used, it remains difficult to predict their effects on long-term stabilization. While studies in this area have qualitatively described how nasal tip rotation changes over time, they have not quantitatively revealed the stabilization dynamics—when and how quickly it occurs.

Artificial intelligence tools are increasingly used to address challenges across medicine. Clinically relevant applications include machine learning, deep learning, robotics, expert systems, facial recognition, and natural language processing. Machine learning uses structured data and statistical algorithms to identify patterns, classify observations, and predict outcomes [10]. In facial plastic surgery and rhinoplasty, previous studies have focused mainly on outcome simulation, surgeon-style learning, classification of preoperative and postoperative images, automated morphometric assessment, and prediction of patient satisfaction [10,11,12,13,14]. Although these approaches have advanced image-based assessment, few studies have examined the exploratory use of serial postoperative photogrammetric measurements for dynamic prediction of a defined 12-month anatomical outcome.

Representative studies on postoperative nasal tip stability and artificial intelligence or machine learning in rhinoplasty are summarized in Table 1. Collectively, the literature has described technique-specific changes in rotation and projection and has explored image-based outcome assessment, but it has not established whether serial postoperative NLA measurements can provide a dynamically updated estimate of the 12-month angle or patient-specific residual loss.

The primary objective of this study was to predict the absolute NLA at postoperative month 12 using measurements available at earlier follow-up visits. Secondary objectives were to characterize the longitudinal course of NLA from the preoperative assessment through month 12 and to quantify residual angle loss from postoperative months 1, 3, and 6 to month 12.

## 2. Materials and Methods

### 2.1. Study Design and Participants

This study was designed as a single-center, retrospective, observational cohort study. The patient records and standard photographic archives of individuals who underwent primary open rhinoplasty using an open approach by a single surgeon at the Otorhinolaryngology Clinic of Amasya University Sabuncuoğlu Şerefeddin Training and Research Hospital between January 2023 and May 2025 were retrospectively reviewed. The study was approved by the Amasya University Non-Interventional Clinical Research Ethics Committee (decision no. 2026/135, 13 July 2026) and was conducted in accordance with the Declaration of Helsinki. Patients were eligible if they were aged 18 years or older, underwent primary open rhinoplasty by the same surgeon, had no history of nasal surgery, and had complete standardized lateral photographs obtained preoperatively and at postoperative months 1, 3, 6, and 12. Exclusion criteria were revision rhinoplasty, concurrent facial aesthetic or maxillofacial surgery, significant facial trauma or nasal fracture history, congenital craniofacial deformity, marked facial asymmetry, prior nasal filler injections, incomplete follow-up photography, and inadequate positional standardization. During the study period, 150 patients underwent primary open rhinoplasty. Of these, 72 were excluded: 25 because complete photographs at all five time points were unavailable, 17 because of marked facial asymmetry, 21 because of a history of maxillofacial trauma and nasal fracture, and 9 because of prior nasal filler injections. The final cohort comprised 78 patients, providing 390 photographs across five time points. All images were anonymized.

### 2.2. Surgical Techniques and Standardization of Photography

All patients underwent standard open technique primary rhinoplasty under general anesthesia by the same surgeon. A midcolumellar inverted V incision was made and connected with marginal incisions. The skin flap was elevated in the supraperichondrial and subperiosteal planes. Septoplasty was performed, and a septal cartilage graft was harvested for use in later stages. Cartilage and bone hump resection was performed. Medial, transverse, and lateral osteotomies were made. Spreader grafts or flaps were used in the middle-vault. Cephalic resection of the lateral crura was performed, and the same standard nasal tip shaping sutures (dome equalization suture, hemitransdomal suture, interdomal suture) were used. A columellar strut graft was used and secured between the medial crura using a medial crural fixation suture. Subsequently, a septocolumellar suture was used. Finally, the skin flap was closed, skin sutures were placed, and internal and external splints were applied to complete the surgery. Photographs were taken in the clinical photo studio with the same camera and equipment, with the Frankfort horizontal plane parallel to the ground, at a fixed focal length, fixed patient–camera distance, and fixed flash settings. All periods were obtained according to the same protocol. Written consent was obtained from the patients for the use of their photographs for scientific purposes. Representative serial photographs obtained with this protocol are presented in Figure 1.

### 2.3. Anatomical Reference Points and Nasolabial Angle Measurement

Measurements were performed using a semi-automatic image analysis system developed in Python (version 3.12.13) with the OpenCV and NumPy libraries. Analyses were conducted on standardized right lateral profile photographs. For the primary longitudinal dataset of 390 photographs, each of two physicians independently marked the seven anatomical landmarks twice on every image. The mean coordinates of the repeated markings were used as the final landmark coordinates. Images were assigned coded identifiers and presented in randomized order, with both observers blinded to patient identity, follow-up time point, and each other’s measurements. NLA was calculated automatically as the angle between the infratip breakpoint–subnasale and subnasale–labialis superius lines (Figure 2). 

Only these three landmarks were required for NLA calculation. The remaining four landmarks—glabella, nasion, nasal tip, and alar crease—were recorded for anatomical context and additional photogrammetric measurements initially considered but not included in this study. The analyses were based solely on NLA measurements. For the reliability substudy, 40 images were selected using stratified random sampling, comprising eight images from each of the five assessment time points and one image per patient. The two measurement sessions were separated by at least four weeks, and each observer’s previous measurements were concealed during repeat assessment. The four resulting NLA measurements per image were retained for intraobserver and interobserver reliability analyses. The complete sequence from patient selection to clinical interpretation is summarized in Figure 3.

### 2.4. Outcome Variables

The primary outcome and principal prediction target was the absolute NLA measured at postoperative month 12 (NLA_12_). Secondary outcomes were longitudinal changes in NLA across the five assessment time points and residual angle loss from postoperative months 1, 3, and 6 to month 12. For clinical interpretation, expected residual loss at a given follow-up visit was derived from the predicted 12-month value as follows:

$${\hat{\Delta }}_{t-12}={\mathrm{NLA}}_{t}-{\hat{\mathrm{NLA}}}_{12}$$

Here, ${\hat{\Delta }}_{t-12}$ is the predicted residual loss, ${\mathrm{NLA}}_{t}$ is the measured angle at postoperative month *t*, and ${\hat{\mathrm{NLA}}}_{12}$ is the predicted month-12 angle. Positive values indicated a predicted decrease in angle between the relevant follow-up visit and month 12.

### 2.5. Statistical and Prediction Analyses

Continuous variables are presented as mean ± standard deviation and, where appropriate, 95% confidence intervals; categorical variables are presented as counts and percentages. Changes in NLA across the five assessment time points were evaluated using the Friedman test, with Kendall’s W reported as the effect-size measure. Pairwise comparisons were reanalyzed using two-sided Wilcoxon signed-rank tests, with Holm adjustment across the five prespecified pairwise comparisons. Mean and median within-patient differences were reported to describe the magnitude and direction of change. Confidence intervals for mean differences were calculated using the t distribution with 77 degrees of freedom and the standard error of the paired differences. These intervals were not adjusted for multiple comparisons and were reported separately from the Holm-adjusted Wilcoxon *p*-values. All tests were two-sided, and *p* < 0.05 was considered statistically significant.

Intraobserver and interobserver reliability were quantified using ICC(2,1), a two-way random-effects, single-measure, absolute-agreement model. Ninety-five percent confidence intervals were obtained from 10,000 image-level bootstrap resamples. Agreement was further assessed using Bland–Altman analysis, with differences calculated as the first measurement minus the second. Mean bias, the standard deviation (SD) of paired differences, and the 95% limits of agreement (LoA; bias ± 1.96 × SD) were reported. The standard error of measurement (SEM) was calculated as the square root of the residual mean square, and the minimum detectable change at the 95% confidence level (MDC95) was calculated as 1.96 × √2 × SEM.

The 12-month nasolabial angle was predicted using information sets available at month 1, months 1 + 3, and months 1 + 3 + 6. The prespecified predictor set for the multivariable models consisted of PREOP, POSTOP_1, POSTOP_3, POSTOP_6, and sex; no data-driven feature-selection step was applied. Linear regression, Ridge, Elastic Net, SVR-RBF, random forest, Extra Trees, and Gradient Boosting algorithms were compared. To assess whether multivariable modeling added predictive value beyond simple methods, three reference models were evaluated for the month-6 information set: an intercept-only model predicting the mean NLA_12_ in the corresponding training fold, a persistence model setting predicted NLA_12_ equal to NLA_6_, and a one-predictor linear regression using NLA_6_ alone. All models used identical patient-level outer splits. Five-repeated 10-fold nested cross-validation was used, with patients rather than photographs assigned to folds. The outer resampling used RepeatedKFold with n_splits = 10, n_repeats = 5, and random_state = 42. Within each outer training set, hyperparameters were tuned using shuffled five-fold cross-validation with random_state = 1000 + split number and MAE as the selection criterion. Standardization and tuning were confined to the corresponding training folds. Regression performance was summarized from held-out predictions using MAE, RMSE, and R^2^. For uncertainty estimation and paired comparisons, each patient contributed the mean of their five held-out absolute errors. Patient-level 95% confidence intervals for MAE and paired MAE differences were obtained from 20,000 bootstrap resamples using random seed 20260827 and the percentile method. Paired differences were defined as Elastic Net MAE minus comparator MAE; negative values favored Elastic Net. Repeated folds were not treated as independent observations. The Ridge search grid comprised 33 alpha values logarithmically spaced from 10^−4^ to 10^4^. For Elastic Net, 31 alpha values from 10^−4^ to 10^2^ were crossed with l1_ratio values of 0.05, 0.10, 0.20, 0.30, 0.50, 0.70, 0.90, 0.95, and 1.00. Algorithms without a prespecified model-specific search grid were fitted using the settings reported in the analysis code. Permutation importance was calculated only after fitting within each outer training fold and was evaluated on the corresponding held-out observations.

## 3. Results

After presentation of the cohort characteristics and measurement reliability, the Results report the secondary longitudinal analyses, followed by the primary prediction analysis for the absolute 12-month NLA and the secondary residual-loss analyses.

### 3.1. Patient Characteristics and Secondary Longitudinal Changes in the Nasolabial Angle over Time

The study included 78 patients with measurements available at all five assessment time points. Of these, 65 (83.3%) were women and 13 (16.7%) were men. A total of 390 measurements were analyzed, with no missing or duplicate records at the patient-time-point level. The 72 excluded procedures were not included in the analytic dataset; because basic demographic and clinical variables for these excluded patients were not available in a sufficiently complete and standardized form, an included-versus-excluded characteristics comparison could not be performed.

The mean preoperative nasolabial angle was 88.06 ± 11.91°. It increased to 107.03 ± 8.18° at postoperative month 1 and subsequently decreased to 101.20 ± 7.30° at month 3, 97.75 ± 6.87° at month 6, and 93.74 ± 7.22° at month 12 (Table 2; Figure 4). The change across time points was significant (Friedman chi-square(4) = 219.32; *p* < 0.001), and Kendall’s W = 0.703 indicated a large time effect.

The mean within-patient increase from the preoperative assessment to month 1 was 18.97° (95% CI: 16.62–21.33). This was followed by mean decreases of 5.83° between months 1 and 3, 3.45° between months 3 and 6, and 4.01° between months 6 and 12. At month 12, the angle remained, on average, 5.68° above the preoperative value (95% CI: 3.53–7.83). All five comparisons were significant using two-sided Wilcoxon signed-rank tests with Holm adjustment (all *p* < 0.001; Table 3).

### 3.2. Intraobserver and Interobserver Measurement Reliability

The reliability analysis included 40 images from 40 different patients, with eight images representing each assessment time point. All four comparisons showed excellent absolute agreement. Intraobserver intraclass correlation coefficients (ICC) were 0.990 (95% confidence interval (CI), 0.981–0.994) for Observer 1 and 0.984 (95% CI, 0.966–0.991) for Observer 2. Interobserver ICCs were 0.987 (95% CI, 0.974–0.992) at the first measurement session (T1) and 0.987 (95% CI, 0.974–0.993) at the second session (T2) (Table 4).

Bland–Altman analysis showed small mean biases, ranging from −0.24° to +0.16°, indicating no marked systematic difference within or between observers. The 95% limits of agreement (LoA) were −3.49° to 3.23° for Observer 1, −4.53° to 4.05° for Observer 2, −3.73° to 4.05° for interobserver agreement at T1, and −3.75° to 3.85° for interobserver agreement at T2. Standard error of measurement (SEM) values ranged from 1.21° to 1.55°, and minimum detectable change at the 95% (MDC95) values ranged from 3.36° to 4.29° (Table 5; Figure 5).

### 3.3. Secondary Outcome: Nasolabial Angle Loss

The mean nasolabial angle loss was 13.29 ± 6.98° from postoperative month 1 to month 12, 7.46 ± 6.01° from month 3 to month 12, and 4.01 ± 4.16° from month 6 to month 12.

### 3.4. Primary Analysis: Prediction of the 12-Month Nasolabial Angle and Secondary Residual-Loss Analysis

In the dynamic prediction of the 12-month nasolabial angle, the lowest MAE using data available through month 1 was 4.53° with the SVR-RBF model. Adding POSTOP_3 reduced the MAE to 4.38°. When POSTOP_6 was included, Elastic Net achieved an MAE of 3.47°, an RMSE of 4.25°, and an R^2^ of 0.650 (Table 6; Figure 6). The additional benchmark analysis presented in Section 3.5 was performed to determine whether this performance represented added value beyond simpler month-6 prediction strategies.

In the sensitivity analysis that directly modeled rotation loss, the loss in nasolabial angle from month 3 to month 12 was predicted with limited accuracy by linear regression (MAE = 4.65°, R^2^ = 0.138). For loss from month 6 to month 12, the Elastic Net model did not outperform the mean baseline model (MAE: 3.37° vs. 3.34°; Table 7). These findings indicate that although the absolute 12-month nasolabial angle could be predicted, the patient-specific magnitude of rotation loss could not be reliably estimated using the available variables.

### 3.5. Primary Prediction Analysis: Comparison of Regression Algorithms and Variable Importance

Among the multivariable candidate algorithms using data available through month 6, Elastic Net achieved an MAE of 3.47°, an RMSE of 4.25°, and an R^2^ of 0.650. Full linear regression and Ridge produced nearly identical results, whereas the nonlinear models showed higher prediction errors (Table 8; Figure 7).

In the simple-benchmark analysis, Elastic Net performed better than the training-fold mean and persistence models, with paired MAE differences of −2.25° (95% CI, −3.21 to −1.34) and −1.38° (95% CI, −2.13 to −0.61), respectively. However, the one-predictor linear regression using NLA_6_ alone had a slightly lower MAE than Elastic Net (3.35° vs. 3.47°). The paired difference was 0.12° (95% CI, −0.06 to 0.30), indicating no additional predictive value from Elastic Net over the one-predictor model (Table 9).

In the permutation analysis, shuffling POSTOP_6 increased the MAE by an average of 2.71°, identifying it as the strongest predictor. PREOP and POSTOP_3 made limited contributions, whereas the independent contributions of POSTOP_1 and sex were close to zero (Figure 8).

## 4. Discussion

This study describes the 12-month course of the nasolabial angle after rhinoplasty, formally evaluates measurement reliability, and examines the ability of machine learning to predict late outcomes from serial measurements. The nasolabial angle peaked at month 1, decreased at subsequent follow-up visits, and remained above the preoperative level at month 12. Although measurements obtained through month 6 moderately predicted the absolute 12-month angle, patient-specific residual angle loss could not be predicted reliably.

### 4.1. The Course of the Nasolabial Angle over Time

In the cohort, NLA increased from 88.06° preoperatively to 107.03° at 1 month and decreased to 93.74° at 12 months. The strong time effect and significant change in all consecutive periods indicate that early postoperative tip rotation does not represent the final outcome. Resolution of edema, scar maturation, cartilage memory, and soft tissue redraping may contribute to this regression [2,7,9].

The nasolabial angle loss at months 1–12 in this study (13.29°) was higher than that reported in some series examining various types of suture and graft techniques [2,6,8,15]. This difference may be due to the heterogeneity of initial deformity, skin-soft tissue characteristics, and measurement times. Although all procedures were performed by the same surgeon, the absence of graft and suture details as structured variables prevented a direct investigation of this possibility.

The observation of an average additional decrease of 4.01° from month 6 to month 12, and the persistence of the decrease in 80.8% of patients, indicates that the 6-month photograph does not represent the final result for every patient. However, a loss of ≥10° was observed in only 7.7% of cases during this period; it appears that late change is common but limited in most patients.

### 4.2. Prediction Performance and Clinical Interpretation

Prediction of the absolute 12-month angle improved after POSTOP_6 was included. Elastic Net achieved an MAE of 3.47° and an R^2^ of 0.650, and POSTOP_6 was the strongest variable in the permutation analysis. However, the benchmark analysis showed that a linear regression model using NLA_6_ alone achieved a comparable MAE of 3.35°. The paired MAE difference between Elastic Net and the one-predictor model was 0.12° (95% CI, −0.06 to 0.30), providing no evidence of additional predictive value from the multivariable Elastic Net model. Thus, the useful predictive information was derived primarily from the latest serial measurement rather than algorithmic complexity or the addition of earlier measurements and sex.

By contrast, performance was poor when the magnitude of rotation loss was modeled directly. Although the absolute POSTOP_12 value largely retained the level observed at month 6, transforming the target into the POSTOP_6 − POSTOP_12 difference narrowed its variance and increased the relative influence of measurement error and individual variation in healing. Therefore, predicting the 12-month angle with reasonable accuracy does not imply that the remaining change can be personalized with the same accuracy.

The reliability substudy demonstrated excellent relative reliability, but the Bland–Altman limits and MDC95 provide a more cautious clinical perspective. MDC95 values of 3.36–4.29° and limits approaching ±4° were similar in magnitude to both the Elastic Net MAE of 3.47° and the mean decrease of 4.01° between months 6 and 12. Accordingly, changes of approximately 4° or less in an individual patient may not be clearly distinguishable from measurement variability. This finding helps explain why the absolute 12-month angle was more predictable than patient-specific residual loss and limits the individual-level clinical interpretation of the models.

Artificial intelligence research in rhinoplasty has primarily focused on simulation, aesthetic scoring, and surgical planning [10,13]. The present study differs by relating serial postoperative measurements to a continuous, time-dependent outcome. Nevertheless, internal cross-validation cannot replace external validation; model reporting and future validation should follow the TRIPOD + AI and PROBAST + AI principles [16,17,18].

The findings should be interpreted in light of several limitations. The single-center retrospective design, sample of 78 patients, and predominance of women should be considered when generalizing the results to other populations. The absence of structured data on surgical technique, graft and suture types, skin thickness, baseline nasal morphology, and related clinical variables limited assessment of their potential effects on NLA stabilization. Measurements were based on two-dimensional lateral photographs, and although a formal two-observer reliability analysis was performed, it was limited to a 40-image sample and does not address the inherent limitations of two-dimensional photogrammetry. The analysis was also restricted to NLA. The sample size limits the precision of performance estimates, and repeated cross-validation does not increase the effective sample size. Nevertheless, complete 12-month follow-up at standardized time points for all patients strengthened the longitudinal analysis. Future larger, prospective, multicenter studies should expand reliability assessment, incorporate surgical and patient-specific variables, and relate geometric findings to patient satisfaction, functional outcomes, and revision requirements.

## 5. Conclusions

After rhinoplasty, the nasolabial angle reached its highest level at postoperative month 1 and decreased significantly through month 12. Despite a mean loss of 13.29° between months 1 and 12, the 12-month value remained, on average, 5.68° above the preoperative level. The angle continued to decrease after month 6 in most patients, although the mean additional loss was limited to approximately 4°.

The month-6 measurement showed moderate internally validated ability to estimate the absolute 12-month NLA. Elastic Net outperformed the intercept-only and persistence benchmarks but did not outperform simple linear regression using NLA_6_ alone. More complex multivariable algorithms therefore provided no meaningful additional predictive value in this cohort. Patient-specific rotation loss from months 6 to 12 remained unpredictable using the available variables, and measurement uncertainty further limited individual-level interpretation.

## Figures and Tables

**Figure 1:**
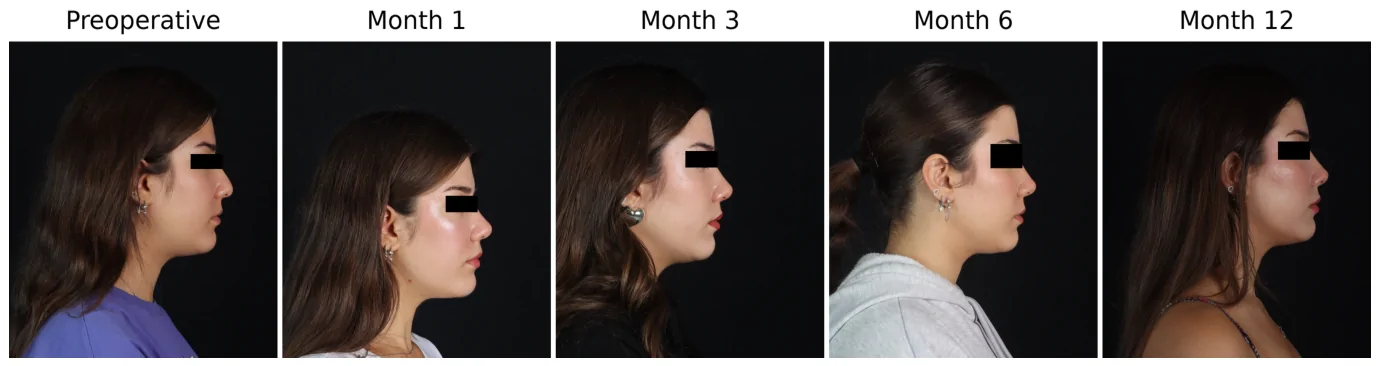
Preoperative and postoperative longitudinal photogrammetric measurement panel for a patient at 1, 3, 6, and 12 months.

**Figure 2:**
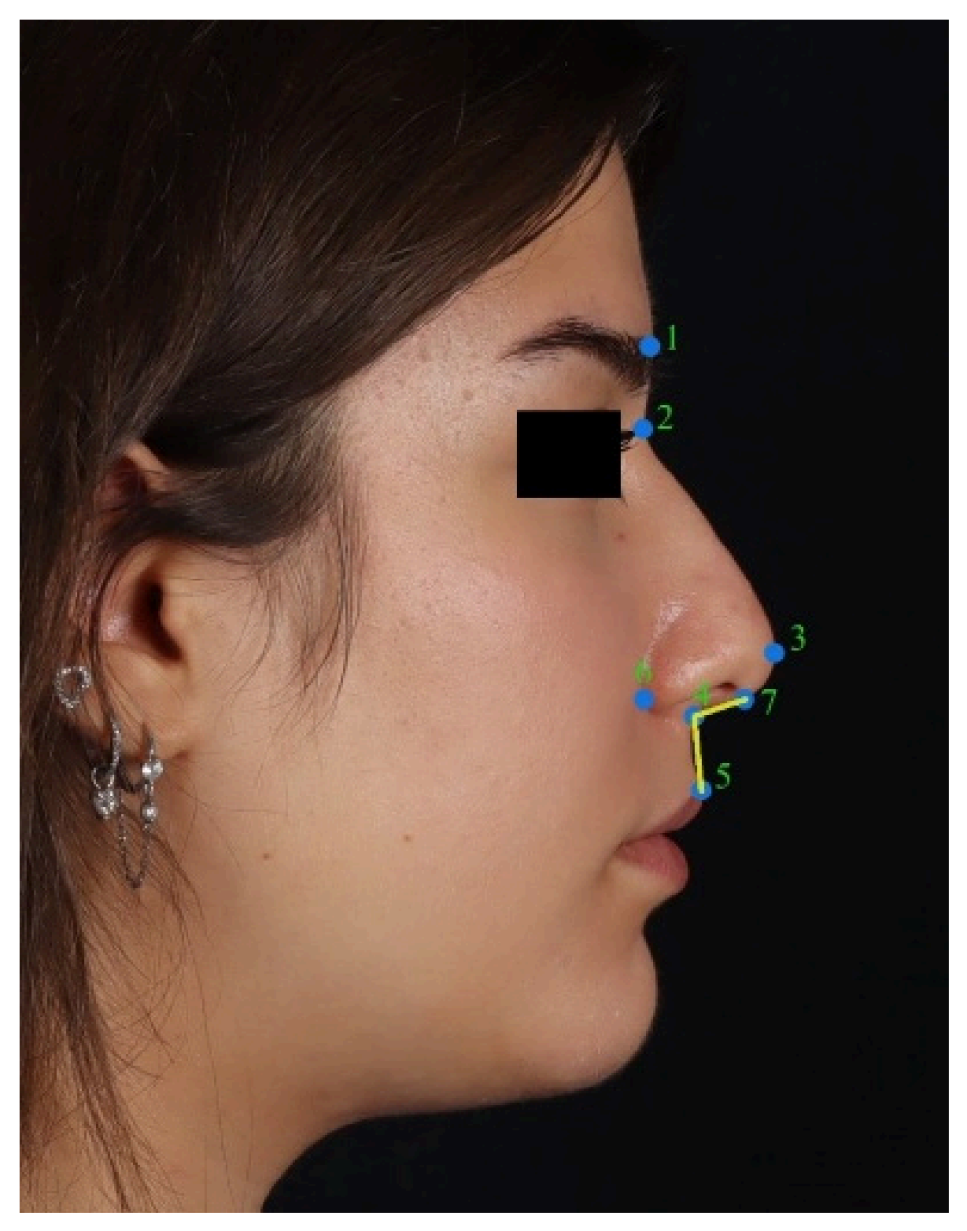
Representative illustration of anatomical reference points and nasolabial angle measurement. (1) glabella, (2) nasion, (3) nasal tip, (4) subnasale, (5) labialis superior, (6) alar crease, (7) infratip break point.

**Figure 3:**
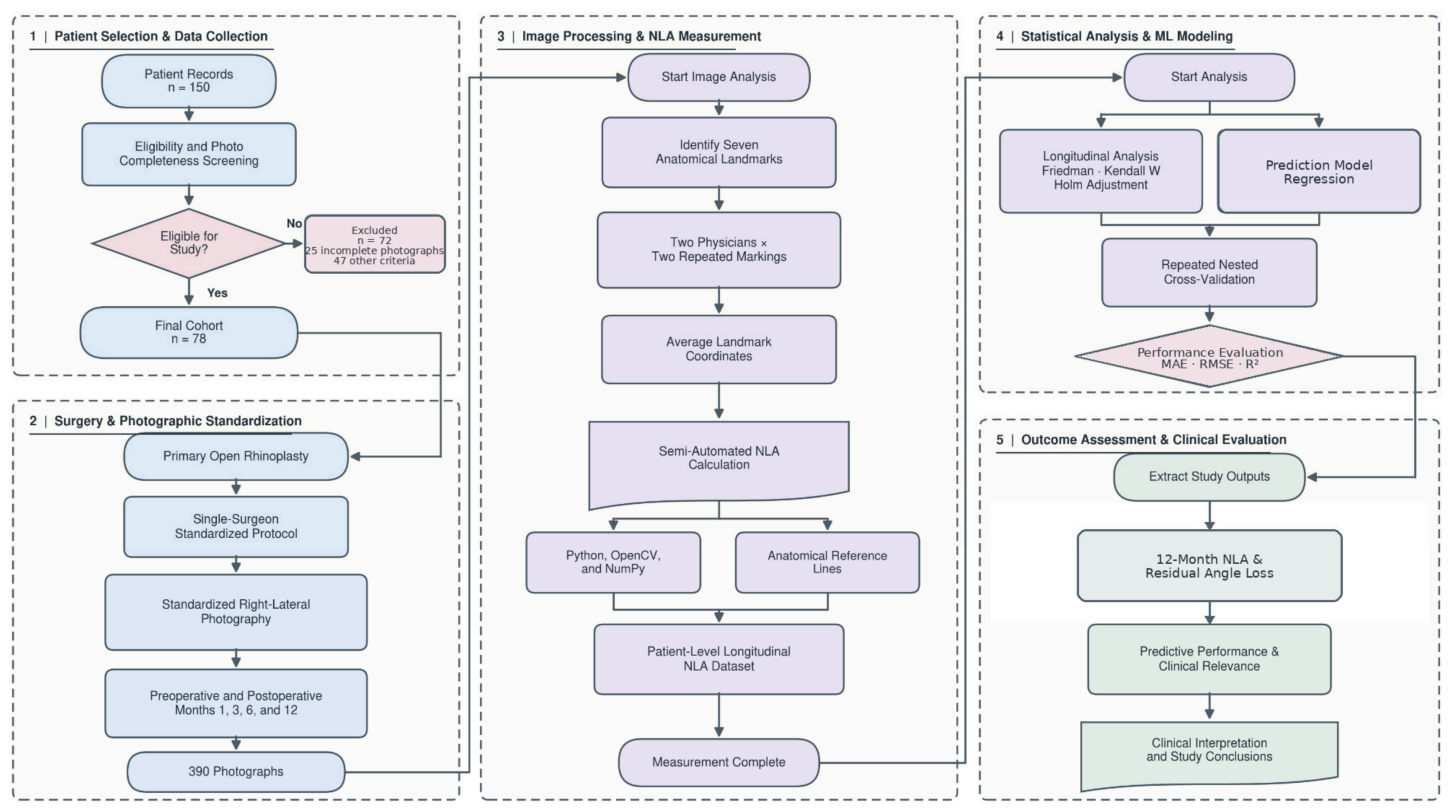
Study workflow from patient selection and standardized photography through NLA measurement, statistical analysis, machine-learning modeling, and clinical interpretation. The cohort flow distinguishes 25 exclusions for incomplete photographs from 47 exclusions meeting one or more other criteria. Two physicians each performed two landmark placements per image; reliability was assessed in a 40-image subset.

**Figure 4:**
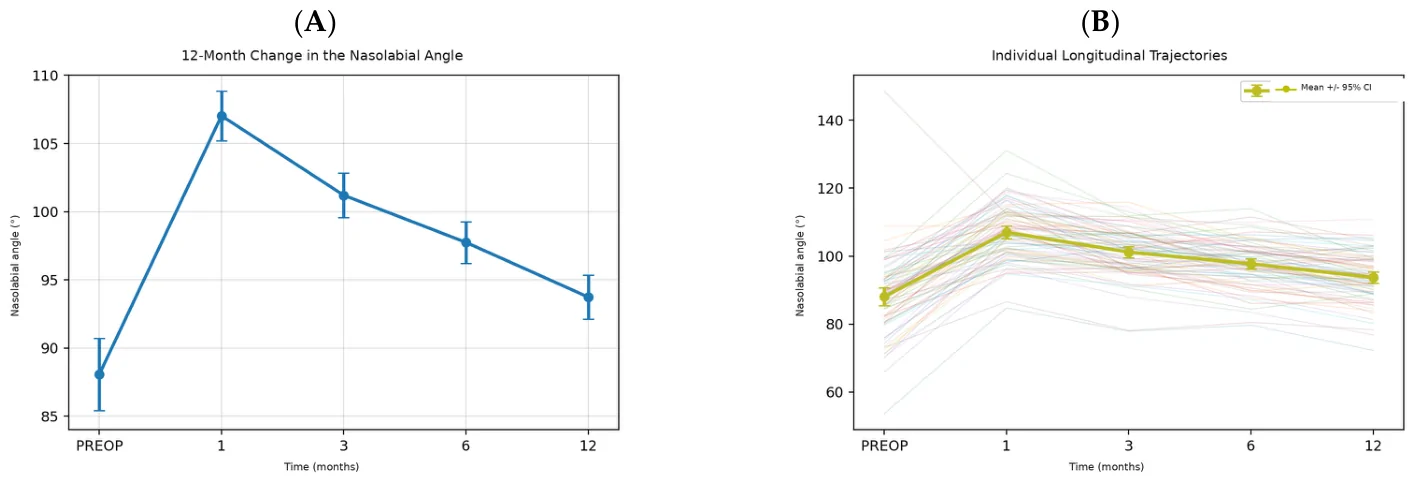
Change in the nasolabial angle from the preoperative period to month 12. (**A**) Mean values and 95% confidence intervals. (**B**) Individual patient trajectories and the group mean.

**Figure 5:**
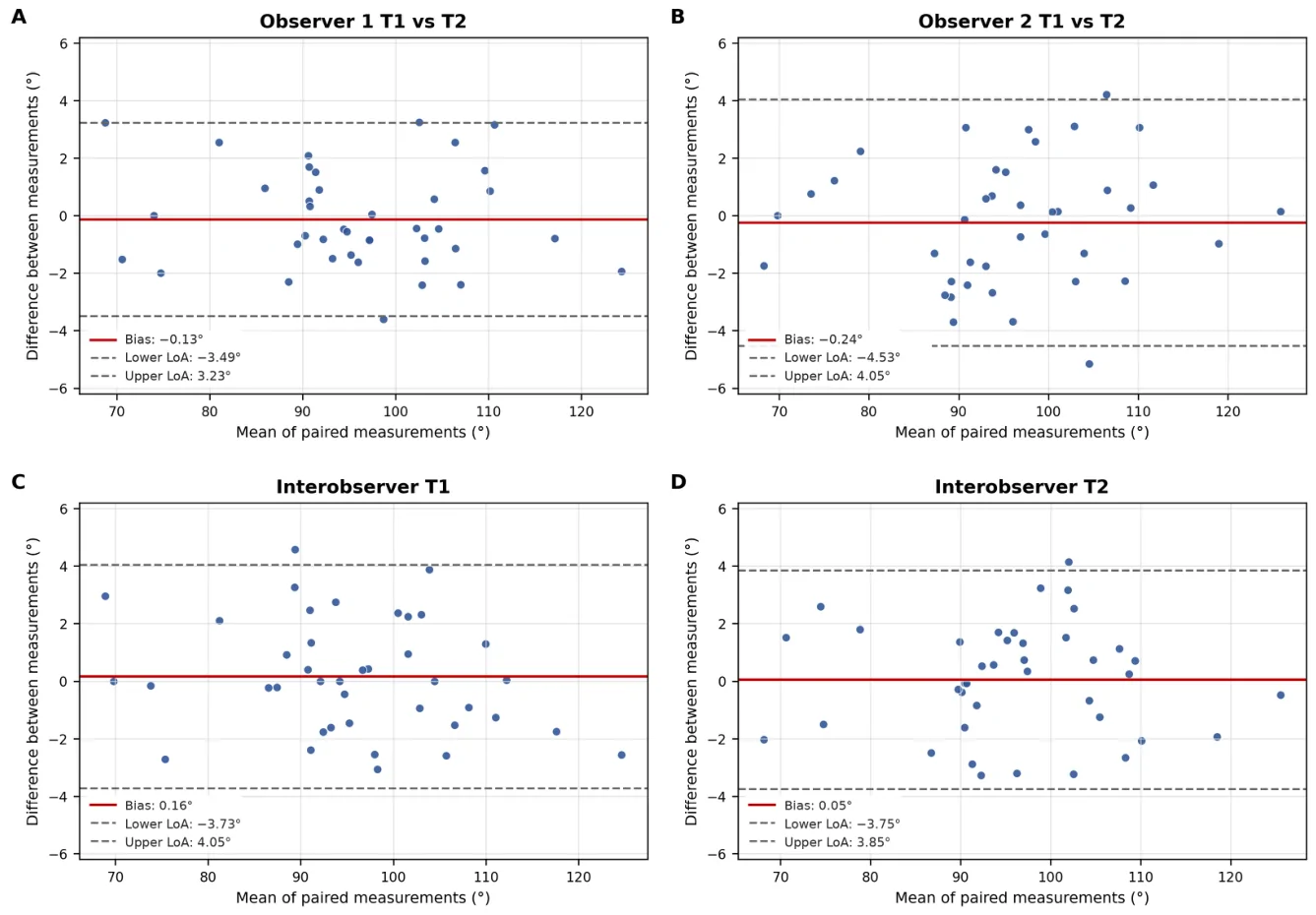
Bland–Altman plots for NLA measurement reliability. (**A**) Observer 1, T1 versus T2; (**B**) Observer 2, T1 versus T2; (**C**) interobserver agreement at T1; and (**D**) interobserver agreement at T2. Solid red lines indicate mean bias; dashed gray lines indicate the 95% limits of agreement.

**Figure 6:**
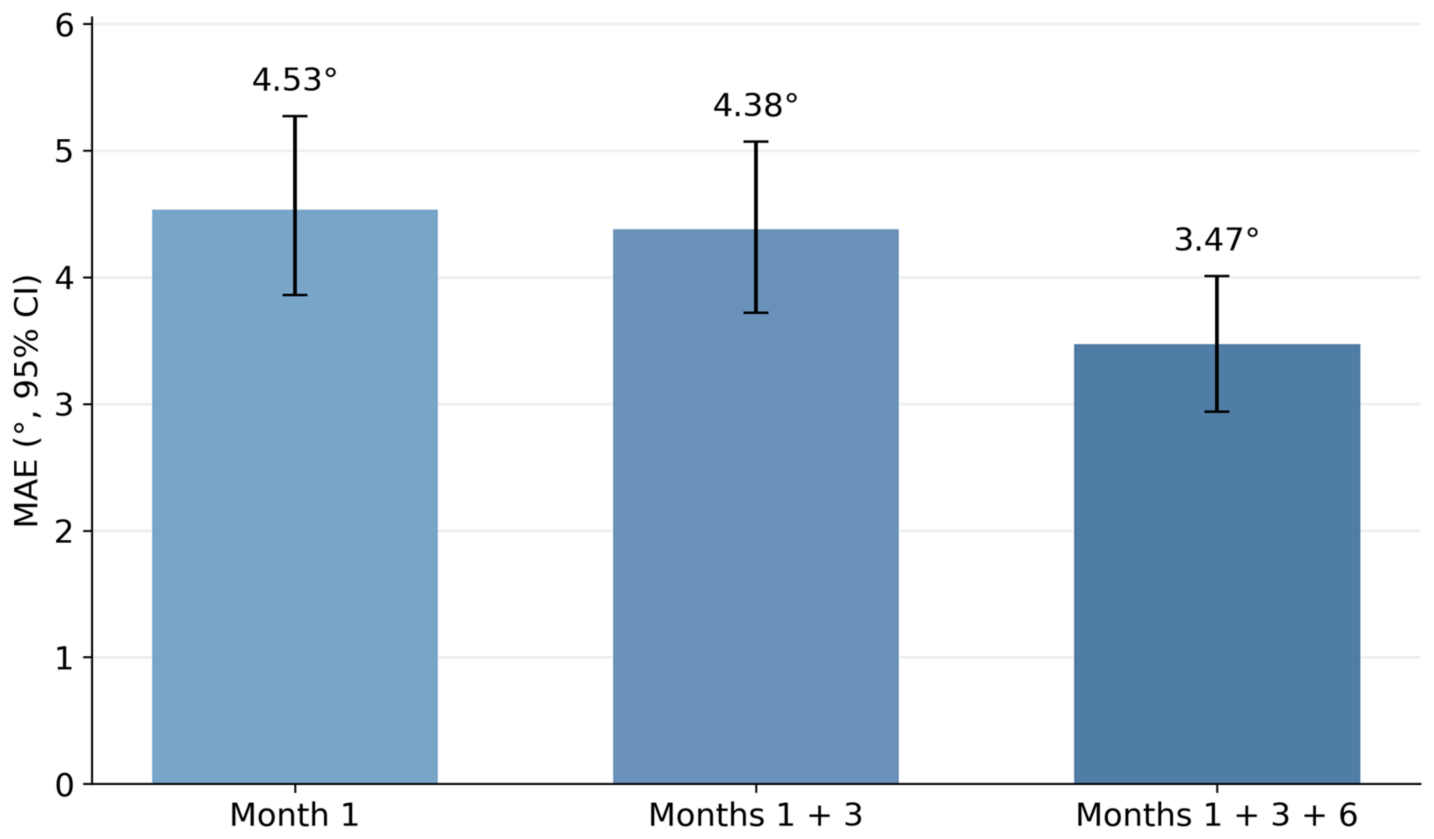
Change in cross-validated 12-month prediction error as early postoperative information increased.

**Figure 7:**
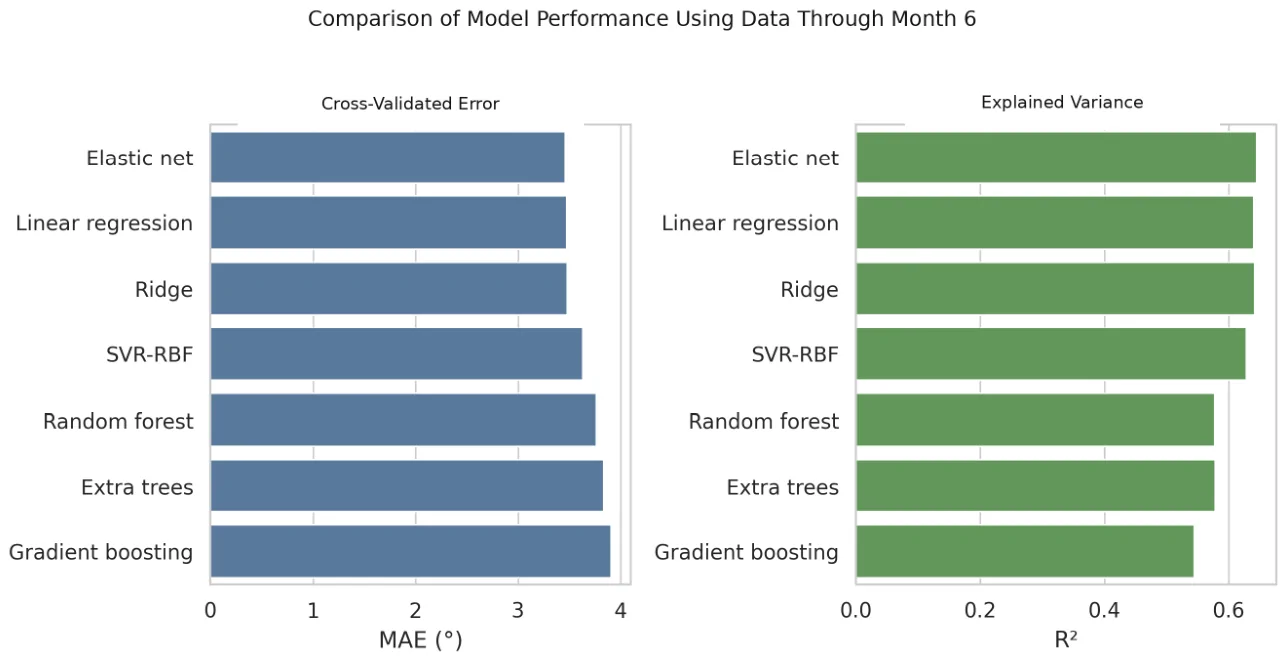
Cross-validated performance comparison of models using data available through month 6.

**Figure 8:**
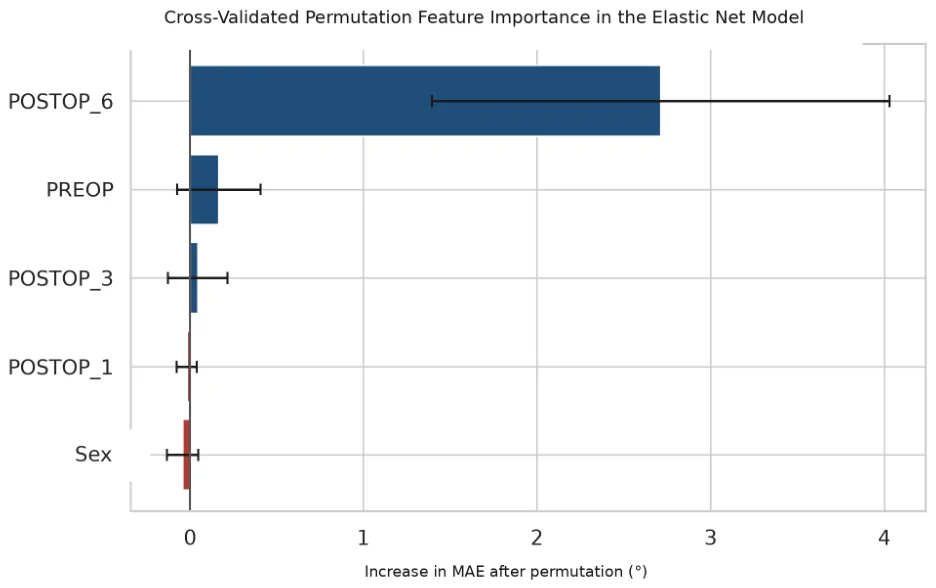
Cross-validated permutation feature importance in the final Elastic Net model.

**Table 1:** Representative studies on nasal tip stability and AI/ML applications in rhinoplasty.

Study	Design/Approach	Primary Focus	Relevance to the Present Study
Çelik et al., 2025 [1]	Clinical technique study	Columellar auto-strut and tip stabilization	Technique-specific stabilization; no dynamic outcome prediction
Şahin et al., 2026 [2]	Longitudinal clinical study	Pentagonal tip support; rotation and projection stability	Serial postoperative stability; technique-specific cohort
Bucher et al., 2020 [3]	Retrospective cohort	Columellar strut effects on NLA and projection	Demonstrated technique-related changes; no individualized late-loss model
Aksakal, 2021 [5]	Comparative clinical study	Tongue-in-groove versus septocolumellar suture/strut	Compared support methods using rotation and projection outcomes
Şimşek et al., 2026 [6]	Clinical cohort	Anterior extended suture with columellar strut	Focused on NLA stability after a defined technique
Mahmoud et al., 2025 [7]	Systematic review and meta-analysis	Columellar strut versus septal extension graft	Synthesized long-term rotation/projection outcomes; no patient-level prediction
Şirinoğlu, 2017 [8]	Clinical cohort	Short floating strut and septocolumellar suture	Evaluated postoperative tip rotation and projection
Pou et al., 2022 [9]	Clinical cohort	Preservation of tip rotation and projection	Assessed longitudinal preservation after open septorhinoplasty
Antunes and Quatela, 2018 [15]	Clinical cohort	Tongue-in-groove maneuver	Evaluated postoperative change in tip rotation
Eldaly et al., 2022 [10]	Systematic review	Simulation and artificial intelligence in rhinoplasty	Mapped AI applications; limited evidence on serial anatomical prediction
Dorfman et al., 2020 [11]	Methodological perspective	Machine learning and objective rhinoplasty assessment	Highlighted objective assessment opportunities
Topsakal et al., 2023 [13]	Machine-learning outcome study	Objective assessment of rhinoplasty outcomes	Direct ML application; not focused on serial 12-month NLA prediction
Varghaei et al., 2025 [12]	Automated image-analysis study	Aesthetic outcomes in facial plastic surgery	Automated outcome assessment; not a longitudinal NLA model

NLA, nasolabial angle; AI, artificial intelligence; ML, machine learning. This table is a representative narrative summary rather than a systematic review.

**Table 2:** Descriptive statistics for nasolabial angle measurements.

Time	*n*	Mean ± SD (°)	95% CI	Median (IQR)	Min–Max
Preoperative	78	88.06 ± 11.91	85.42–90.70	88.94 (82.05–93.39)	53.65–148.47
Month 1	78	107.03 ± 8.18	105.22–108.85	107.09 (101.38–112.54)	84.79–131.11
Month 3	78	101.20 ± 7.30	99.58–102.82	102.01 (96.54–106.48)	77.88–115.88
Month 6	78	97.75 ± 6.87	96.22–99.27	97.75 (93.84–101.80)	79.71–114.02
Month 12	78	93.74 ± 7.22	92.14–95.34	93.62 (89.23–98.99)	72.27–110.90

SD: standard deviation; CI: confidence interval; IQR: interquartile range. Overall effect of time: Friedman chi-square(4) = 219.32; *p* < 0.001.

**Table 3:** Holm-adjusted paired comparisons between time points.

Comparison	Mean Difference (°)	95% CI	Median Difference (°)	Holm-Adjusted *p*
Preoperative → Month 1	18.97	16.62–21.33	20.04	<0.001
Month 1 → Month 3	−5.83	−7.03 to −4.63	−5.07	<0.001
Month 3 → Month 6	−3.45	−4.62 to −2.29	−3.20	<0.001
Month 6 → Month 12	−4.01	−4.95 to −3.07	−4.20	<0.001
Preoperative → Month 12	5.68	3.53–7.83	6.31	<0.001

Differences were calculated as the later measurement minus the earlier measurement. *p*-values were obtained using two-sided Wilcoxon signed-rank tests with Holm adjustment across the five reported comparisons. The 95% confidence intervals describe mean paired differences, were calculated using the t distribution, and were not adjusted for multiplicity.

**Table 4:** Intraobserver and interobserver reliability of NLA measurements.

Comparison	*n*	ICC(2,1)	95% CI	Interpretation
Observer 1, T1 vs. T2	40	0.990	0.981–0.994	Excellent
Observer 2, T1 vs. T2	40	0.984	0.966–0.991	Excellent
Interobserver, T1	40	0.987	0.974–0.992	Excellent
Interobserver, T2	40	0.987	0.974–0.993	Excellent

Note: ICC, intraclass correlation coefficient; CI, confidence interval. ICCs were estimated using a two-way random-effects, single-measure, absolute-agreement model.

**Table 5:** Bland–Altman agreement and measurement-error estimates for NLA measurements.

Comparison	Bias (°)	SD (°)	Lower LoA (°)	Upper LoA (°)	SEM (°)	MDC95 (°)
Observer 1, T1 vs. T2	−0.13	1.71	−3.49	3.23	1.21	3.36
Observer 2, T1 vs. T2	−0.24	2.19	−4.53	4.05	1.55	4.29
Interobserver, T1	0.16	1.98	−3.73	4.05	1.40	3.89
Interobserver, T2	0.05	1.94	−3.75	3.85	1.37	3.80

Note: Differences were calculated as the first measurement minus the second. LoA, limits of agreement; SEM, standard error of measurement; MDC95, minimum detectable change at the 95% confidence level.

**Table 6:** Performance of the best multivariable candidate model at each information time point.

Information Time Point	Best Model	MAE (°), 95% CI	RMSE (°)	R^2^
Month 1	SVR-RBF	4.53 (3.86–5.27)	5.56	0.401
Months 1 + 3	SVR-RBF	4.38 (3.72–5.07)	5.34	0.447
Months 1 + 3 + 6	Elastic Net	3.47 (2.94–4.01)	4.25	0.650

**Table 7:** Cross-validated prediction of residual angle loss from the month 3 and month 6 follow-up visits to month 12.

**Prediction Time Point**	**Model**	**MAE (°)**	**RMSE (°)**	**R^2^**
Month 3	Mean baseline	4.99	6.04	−0.021
Month 3	Linear regression	4.65	5.55	0.138
Month 6	Mean baseline	3.34	4.19	−0.030
Month 6	Elastic Net	3.37	4.24	−0.053

**Table 8:** Comparison of regression algorithms using data available through month 6.

**Algorithm**	**MAE (°)**	**RMSE (°)**	**R^2^**
Elastic Net	3.47	4.25	0.650
Full linear regression	3.47	4.29	0.643
Ridge	3.49	4.29	0.642
SVR-RBF	3.63	4.38	0.628
Random forest	3.76	4.67	0.577
Extra Trees	3.83	4.66	0.578
Gradient Boosting	3.90	4.84	0.545

**Table 9:** Comparison of Elastic Net with clinically meaningful simple benchmarks using month-6 information.

Model	MAE (°), 95% CI	RMSE (°)	R^2^	ΔMAE: EN − Comparator (°), 95% CI
Training-fold mean	5.72 (4.77–6.74)	7.24	−0.018	−2.25 (−3.21 to −1.34)
Persistence (NLA_12_ = NLA_6_)	4.85 (4.18–5.56)	5.76	0.357	−1.38 (−2.13 to −0.61)
Linear regression (NLA_6_ only)	3.35 (2.80–3.92)	4.18	0.661	0.12 (−0.06 to 0.30)
Elastic Net	3.47 (2.94–4.01)	4.25	0.650	Reference

Note: Performance metrics were calculated from held-out predictions. For paired comparisons and confidence intervals, each patient contributed their mean absolute error across five held-out appearances. ΔMAE was defined as Elastic Net minus comparator; negative values favor Elastic Net. Confidence intervals were obtained from 20,000 patient-level bootstrap resamples and were not adjusted for multiple comparisons.

## Data Availability

The data supporting the findings of this study are not publicly available due to ethical restrictions related to patient privacy. Anonymized data may be made available upon reasonable request to the corresponding author, subject to institutional approval.
